# How New York Physicians and Dentists Get a Competence

**Published:** 1874-06

**Authors:** 


*How New York Physicians and Dentists get a Competence.*—From a New York letter in a daily paper we take the following:

"A physician in good practice will receive patients at his house four hours daily, and make calls for about the same length of time. From ten to twenty callers, and half as many house patients, would be a fair average; the fees would be two and five dollars each. At these figures it would not be hard to make up an income of $20,000 or more. It is said of Dr. Williard Parker, I believe, that having been called out of town to attend a patient, returned a bill of $300, and when it was disputed, he showed by his books that his daily receipts were much over that sum.

Surgeons single charges are much larger than those of physicians, though the income of the latter are probably the highest. For ordinary attendance their rates are about the same, or say $5 a visit. From $25 upward is the charge for operations. For setting an arm or leg $250 would be asked, larger undertakings being in proportion. For a case requiring delicate operation and six weeks' constant attendance, sometimes two or three times a day, $1,000 was lately asked by a leading surgeon. In another instance, where a wealthy gentleman was badly jammed by a railroad car, he was attended by Dr. James R. Wood, who made about a dozen visits, without any important operation, and sent in a bill of $2,500, which was paid.

This is exceeded by Dr. Carnochan, who charged $2,000 for an operation alone, while another surgeon is said to have received $4,500 from one patient. The prices charged by dentist's are quite as high as those of physicians. A man of ordinary reputation in the profession will ask from $5 to $30 for pulling a single tooth, while Dr. Atkinson, one of the most fashionable dentists, is reported to charge $10 for simply examining a person's teeth, and $25 an hour for operating upon them. Many people refuse to pay these fancy prices, but it is a common thing to have to pay from $10 to $100 dentists' bills.

Most practitioners of any reputation have engagements very far ahead. Ten days is a short time to wait for your turn, while a friend of mine, who went to Europe in the middle of last October, on applying to her dentist for treatment, was told that he could not give her a single hour's heed until February, or nearly four months in advance. Dentists are kept busy all the year round, and seldom have any leisure. Their practice is confining and not healthy, but it is very profitable. Their incomes range from $5,000 to $50,000 a year."

*Death while under the Influence of Chloroform.*—Mr. Samuel Hartzel, of No. 166 Pearl Street, Baltimore, died very suddenly while under the influence of chloroform. It seems that about two weeks ago, Mr. Hartzel dislocated his left shoulder, and yesterday his physicians, Drs. Butler and Keller, desiring to perform an operation which they considered necessary, administered chloroform. While Mr. Hartzel was enhaling the vapor, he was seized with a violent spasm, during which he died. Every effort was made to resuscitate him but without success. It was the opinion of the physicians that death resulted from heart disease, as the deceased had been subject to attacks of that affection. As Mr. Hartzel's relatives would not permit a post mortem examination, the coroner declined holding an inquest.

